# Roles of PI3Kγ and PI3Kδ in mantle cell lymphoma proliferation and migration contributing to efficacy of the PI3Kγ/δ inhibitor duvelisib

**DOI:** 10.1038/s41598-023-30148-3

**Published:** 2023-03-07

**Authors:** Kathleen J. Till, Mariah Abdullah, Tahera Alnassfan, Gallardo Zapata Janet, Thomas Marks, Silvia Coma, David T. Weaver, Jonathan A. Pachter, Andrew R. Pettitt, Joseph R. Slupsky

**Affiliations:** 1 Department of Molecular and Clinical Cancer Medicine, Institute of Systems, Molecular and Integrative Biology, University of Liverpool, Ashton Street, Liverpool, L69 3GE UK; 2 Verastem Oncology, 117 Kendrick St #500, Needham, MA 02494 USA

**Keywords:** Haematological cancer, Lymphoma, Cancer, Targeted therapies, Target validation

## Abstract

Mantle cell lymphoma (MCL) is an aggressive B-cell non-Hodgkin lymphoma that is incurable with existing therapies, and therefore presents a significant unmet clinical need. The ability of this disease to overcome therapy, including those that target the B cell receptor pathway which has a pathogenic role in MCL, highlights the need to develop new treatment strategies. Herein, we demonstrate that a distinguishing feature of lymph node resident MCL cells is the expression of phosphatidylinositol 3-kinase γ (PI3Kγ), a PI3K isoform that is not highly expressed in other B cells or B-cell malignancies. By exploring the role of PI3K in MCL using different PI3K isoform inhibitors, we provide evidence that duvelisib, a dual PI3Kδ/γ inhibitor, has a greater effect than PI3Kδ- and PI3Kγ-selective inhibitors in blocking the proliferation of primary MCL cells and MCL cell lines, and in inhibiting tumour growth in a mouse xenograft model. In addition, we demonstrated that PI3Kδ/γ signalling is critical for migration of primary MCL cells and cell lines. Our data indicates that aberrant expression of PI3Kγ is a critical feature of MCL pathogenesis. Thus, we suggest that the dual PI3Kδ/γ duvelisib would be effective for the treatment of mantle cell lymphoma.

## Introduction

MCL is an aggressive B-cell non-Hodgkin’s lymphoma (B-NHL) which is incurable with current treatment options, and overall survival (OS) ranges between 3 and 5 years^1–4^. MCL is genetically characterised by the t11;14 translocation which results in overexpression of cyclin D1. Although the translocation and consequence overexpression of cyclin D1 is not sufficient to induce lymphomagenesis it probably facilitates the process by deregulating the cell cycle^1–4^. It has been long been known that MCL has two distinct clinical entities described as classical (nodal) MCL, and a leukaemic (non-nodal) variant. The malignant lymphocytes of classical MCL express the transcription factor *SOX11* and the BCR has unmutated *IGHV* genes^5–7^ with up to 60% of cases using the same 6 genes within the *IGHV3* and *IHGV4* antigen recognition sequences^8,9^. The leukaemic variant has a more indolent clinical course; cells lack *SOX11* and express mutated *IGHV* genes^5,6,10^. Expression of *TP53* results in an aggressive phenotype^5^ and is found in a higher proportion of leukaemic cases^7^.

The importance of the BCR in the pathogenesis of MCL is highlighted by the fact that signalling through the receptor is significantly upregulated in the lymph node (LN)-resident MCL cells as compared to those found in the peripheral blood. Furthermore, patients with the highest levels of BCR signalling have a significantly poorer prognosis^8,11^. BCR signalling is also upregulated in vitro in MCL cell lines where the most abundant upregulated phosphoproteins are those involved in this signalling pathway^12^. Moreover, in a transgenic mouse model, *SOX11* overexpression in B cells leads to amplified BCR signalling and the development of a MCL-like lymphoma^11^. Therefore, inhibitors of the BCR proximal signalling molecules PI3Kδ (idelalisib) and BTK (ibrutinib and acalabrutinib) have been used to treat MCL. Treatment with idelalisib is effective in other B-NHL, however the response in MCL was poor with a progression-free survival (PFS) of 3.5 months^13^. Ibrutinib and acalabrutinib^1,3,14,15^ have been approved for treatment of MCL however they do not improve PFS, or the overall response rate compared to conventional chemoimmunotherapy (CIT)^4^.

The lymphoma microenvironment (LME) also plays an important role in the pathogenesis of MCL. Signals generated by the LME are important for MCL cell proliferation and survival^16^. MCL lymphocytes model the LME, recruiting cells such as lymphoma-associated macrophages; and T-cells which secrete CD40L and IL-4^17,18^ and induce MCL-cell proliferation^19^. Part of the efficacy of BTK inhibitors has been attributed to the fact that they block signals emanating from the LME as well as from the BCR^9,20–22^. Despite this, signalling from the BCR and the LME play a major role in the resistance/failure of currently available therapies^17,23–26^.

An effective therapeutic strategy for MCL would therefore need to involve more successful targeting of signals from both the LME and the BCR pathway. The dual PI3Kδ/γ inhibitor duvelisib was developed with this in mind^27,28^. PI3Kδ is important for BCR signalling and T-cell activation, whilst PI3Kγ is important for activation of macrophages, and trafficking of myeloid cells^29,30^. Duvelisib has been approved for the treatment of the B-NHLs follicular lymphoma (FL) and chronic lymphocytic leukaemia (CLL) where it has the combined properties of inhibiting tumour cell proliferation and survival, as well as modulating the LME^31^. Herein we show that MCL cells, in contrast to other B-NHL cells, express high levels of PI3Kγ and that the kinase is important for both the proliferation and migration of MCL cells. Our data therefore provide a functional rationale for the treatment of MCL patients with the dual PI3Kδ/γ inhibitor duvelisib.

## Results

### MCL LN strongly express PI3Kγ on the malignant B cells

**Figure 1:**
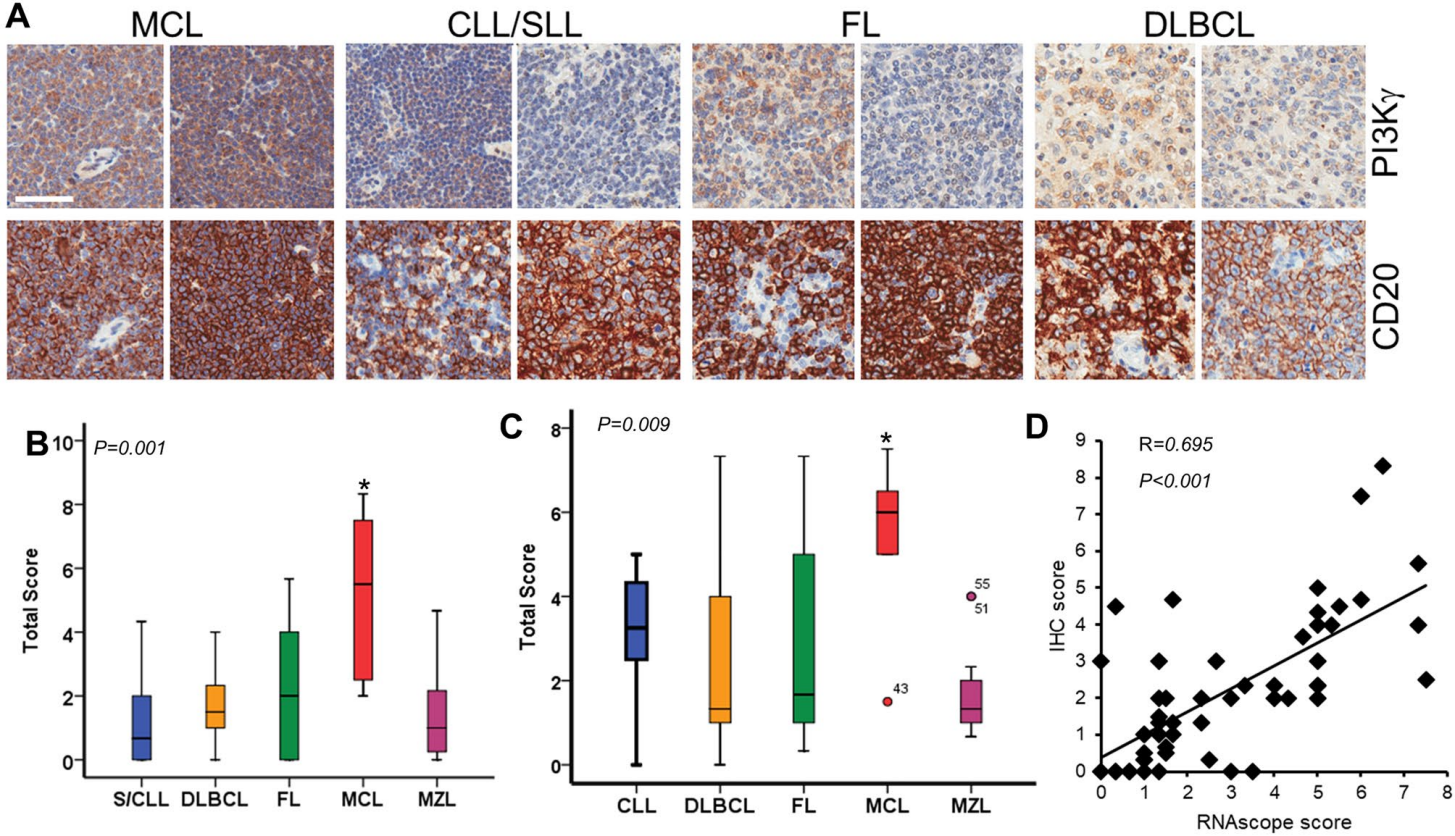
Expression of PI3Kγ within B-NHL in tissues. TMAs were analysed for PI3Kγ expression by IHC and RNAscope using DAB (brown) as the chromogenic substrate; the counter stain was haematoxylin (blue). (**A**) Representative examples of TMA staining for PI3Kγ protein (*upper*) and CD20 (*lower*) on adjacent LN sections taken from patients with MCL, CLL/SLL, FL and DLBCL. For MCL different patterns of positive staining are shown. Whereas, for CLL/SLL, FL and DLBCL the left-hand panel shows cores with high levels of PI3Kγ and the right-hand panel cores with lower levels of staining. Bar = 50 μM. (**B**) Comparison of IHC staining scores. (**C**) Comparison of PI3Kγ mRNA in a serial section of the same TMA measured using RNAScope. *P < 0.05. Statistical significance was determined using the Mann-Witney U-test. (**D**) Correlation, using Pearson’s coefficient, between PI3Kγ protein and mRNA expression as determined by IHC and RNAScope analysis.

We initially undertook to examine expression of PI3Kγ within malignant B cells resident in secondary lymphoid organs (SLO) by immunohistochemistry (IHC) and found PI3Kγ expression in all lymphoma subtypes (representative examples are shown in Fig. 1A). CD20 staining of B cells on the contiguous section indicated that PI3Kγ was found on the malignant lymphocytes as well as in CD20^-^ cells of the LME. PI3Kγ staining was not seen in the CD20 positive areas of the two control normal SLO cores taken from tonsil [(total score ≤ 3 (Supplementary Figure 1A)]. The lymphoma cells of MCL were uniformly positive for PI3Kγ with 83% of LN expressing the kinase (Fig. 1A,B; Supplementary Figure 1B-C). In contrast, the pattern of PI3Kγ staining was variable on the other B-NHL. Thus, only a minority of cases of SLL/CLL (25%), DLBCL (40%), FL (38%) and MZL (25%) expressed PI3Kγ in areas containing CD20^+^ malignant B cells (Fig. 1A,B; Supplementary Figure 1B-C). The malignant B cells from all the B-NHLs had uniform strong expression of PI3Kδ (Supplementary Figure 1D).

We next probed the tissue microarray (TMA) for expression of mRNA coding for PI3KCG (p110γ); mRNA levels were significantly more positive on the malignant lymphocytes of MCL than the other lymphomas analysed (Fig. 1C). A significant positive correlation of PI3Kγ protein and RNA expression was observed (R = 0.695; *P* < 0.001; Fig. 1D). Taken together, these data demonstrate that the lymph node resident MCL cells express PI3Kγ.

Finally, we examined PI3Kγ protein levels on MCL cell lines and peripheral blood mononuclear cells [PBMC (n = 6)]. Similar levels of PI3Kγ were found in cells from the Maver-1 and Mino cell lines, and in purified primary malignant peripheral blood cells from patients with both nodal and leukaemic MCL (Supplementary Figure 1E; patient details Supplementary Table 1). The amount of PI3Kγ on the primary MCL cells, and the Maver-1 and Mino lines was comparable to those of the positive control monocyte cell line (THP-1). However, cells from the Jeko-1 line had a considerably lower amount of the kinase (Supplementary Figure 1E).

### PI3Kγ inhibits the proliferation of MCL cells but does not induce apoptosis

**Figure 2:**
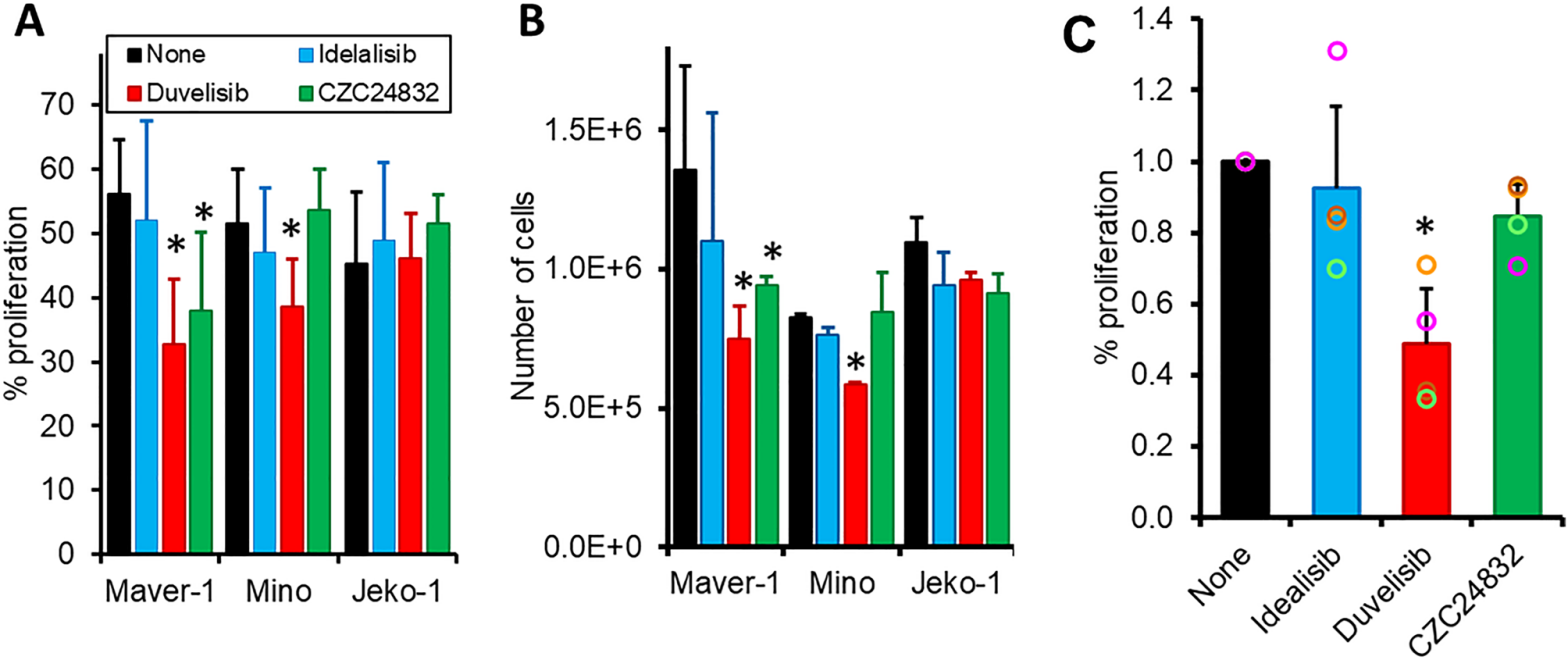
Inhibition of PI3Kγ reduces the proliferation of MCL cells. (**A**, **B**) Maver-1, Mino and Jeko-1 cells were incubated with 1 μM each of the indicated PI3K inhibitors or left untreated for 72 h. 5-ethynyl-2´-deoxyuridine (EdU) was added for the last 4 h of incubation. (**A**) The percentage of proliferating cells which had incorporated EdU into their DNA were determined by FACS analysis. (**B**) The number of cells were also counted manually at 72 h. (**A**, **B** mean ± SD of 3 separate experiments). (**C**) PBMC from MCL patients were stained with CFSE and incubated for 14 d on HS-5 cells in the presence of IL-4; 1 μM each of the indicated PI3K inhibitors was added at the beginning of the culture period. CFSE is diluted proportionally by subsequent cell divisions, and this reduced fluorescence on divided cells was used to calculate the percentage of proliferating MCL cells. Since the levels of proliferation varied between the patients the data is normalised to the untreated control (raw data Supplementary Figure 3D); the bars represent the average proliferation (mean ± SD) and the circles the data for cells from each of the 4 patients studied. *P < 0.05. Statistical significance was determined using the paired Student’s T-Test; for (**C**) the test was performed on the raw data.

In order to explore the functional significance of PI3Kγ expression in MCL malignant cells, we investigated PI3K-dependent cellular properties using MCL cell lines and MCL PBMC. We began by investigating the role of PI3K in the proliferation and apoptosis of MCL cells as the kinases have been shown to play important roles in these processes^32^. To do this we used small molecule inhibitors of PI3Kα (A66), PI3Kδ (idelalisib), PI3Kγ (CZC24832), and the dual PI3Kδ/γ inhibitor (duvelisib). The concentration of inhibitors was determined by inhibition of AKT phosphorylation in response to appropriate stimulation (Supplementary Figure 1F); the concentrations of duvelisib and idelalisib used (1 μM) were below the peak plasma levels of patients treated with the drugs^33,34^. We chose to inhibit these isoforms of PI3K as PI3Kδ inhibition with idelalisib has been shown to be effective in the treatment of B-NHL although the duration of response in MCL was short^13^. Moreover, resistance to kinase inhibitor treatment has been shown, at least in part, to be due to kinome reprogramming involving PI3Kγ^35^. We therefore hypothesised that dual inhibition with the PI3Kγ/δ inhibitor might overcome both primary resistance seen with idelalisib and that induced by ibrutinib treatment. We also included PI3Kα inhibition as the kinase is frequently overexpressed in MCL, and this isoform has also been shown to be involved in ameliorating the effects of idelalisib treatment^36^. Incubation of Maver-1 and Mino cells for up to 72 h with duvelisib caused a significant decrease in the percentage of cells proliferating [*P* < 0.05 (Fig. 2A)]. This was reflected in a significant decrease in the total number of cells [*P* < 0.05; (Fig. 2B)], and in the number of cells in S phase [*P* > 0.05*;* (Supplementary Figure 2A)]. Treatment with CZC24832 had similar effects in Maver-1, but not Mino cells. Treatment with idelalisib or A66 had had no effect on either the number of cells or the percentage of cells proliferating [*P* > *0.05*; (Fig. 2A, Supplementary Figures 2A–C)]. In contrast, the proliferation, cell cycle stage and number of Jeko-1 cells was not affected by incubation with any of the inhibitors [*P* > *0.05;* (Fig. 2A; Supplementary Figures 2A-C)]. The inhibition of proliferation was not due to the induction of apoptosis, as culture with the PI3K inhibitors did not induce apoptosis (Supplementary Figures 3A-C).

We next performed similar experiments on MCL PBMC. Co-culture of MCL PBMC with stromal cells and IL-4 induced proliferation in all cases at 14 days. However, the amount of proliferation varied between cases (n = 4; Supplementary Figure 2D). Treatment with duvelisib significantly reduced proliferation of MCL PBMC in all cases, whereas the effects of idelalisib and CZC24832 were less marked, and not significant when taking the patient group as a whole (Fig. 2C and Supplementary Figure 2D). Inhibition of PI3Kα had no effect (Supplementary Figure 2D). Although apoptosis was observed to a varying degree in all cases, this was not affected by culture with any of the PI3K inhibitors (Supplementary Figures 3D, E).

**Figure 3:**
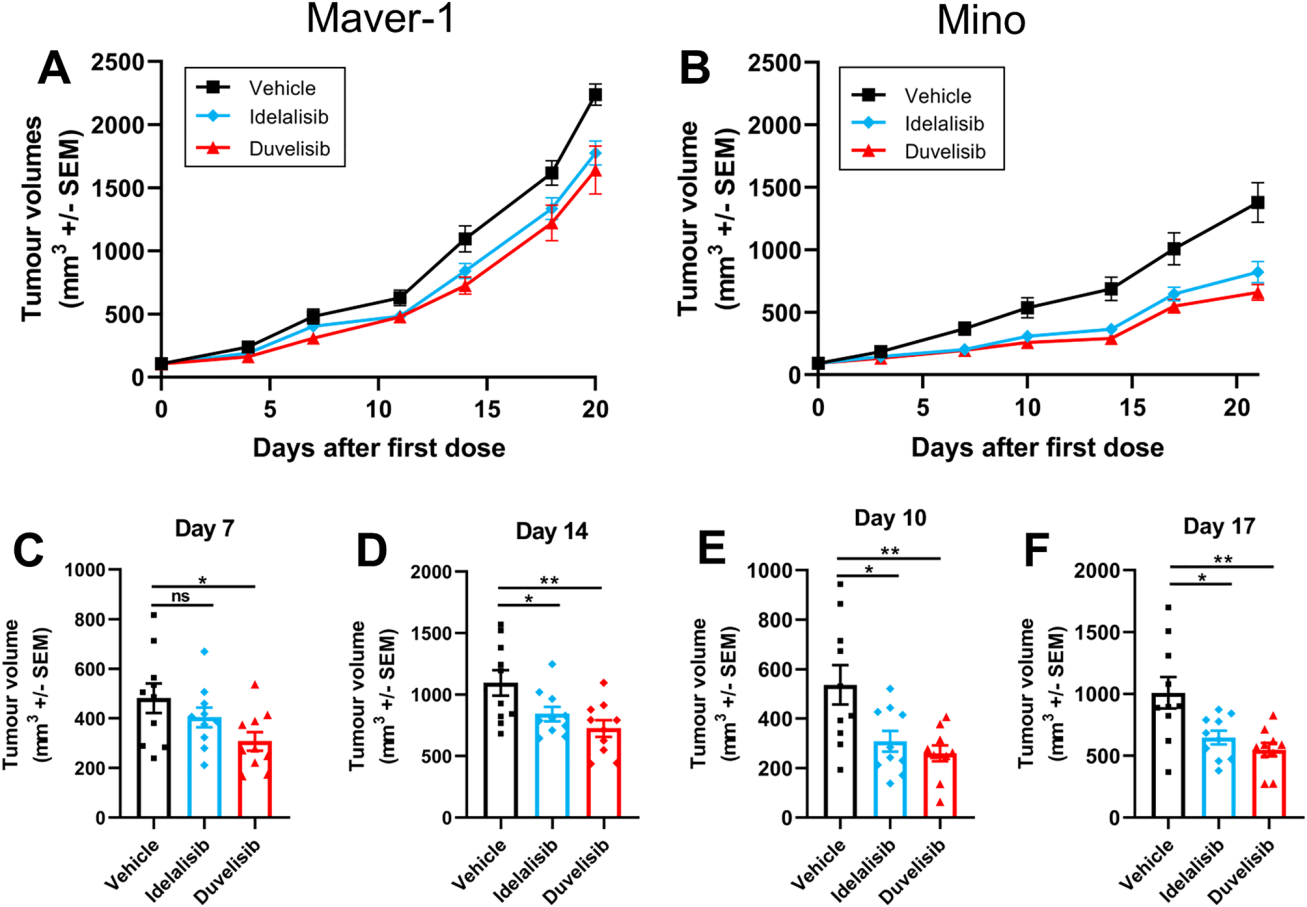
PI3K inhibition reduces tumour growth in a xenograft model. 5 × 10^6^ Maver-1 or Mino cells were injected subcutaneously into SCID mice. When the tumours had reached 60–160 mm^3^ the animals were subjected to either vehicle control or idelalisib/duvelisib at 50 mg/kg. Tumour volumes were measured with a calliper in two dimensions. Tumour volumes of (**A**) Maver-1 and (**B**) Mino cells in engrafted animals over a period of 21 days. (**C**, **D**) Data from xenografts of Maver-1 in the individual mice shown in (**A**) at Day 7 (**C**) and Day 14 (**D**). (**E**, **F**) Data from xenografts of Mino in the individual mice shown in (**B**) at Day 10 (**E**) and Day 17. (**F**) Data in all parts of the figure are presented as mean ± SEM from 10 individual mice. *P < 0.05; **P < 0.001. Statistical significance was determined using the Student’s T-Test.

Finally, we examined the effect of duvelisib and idelalisib on the growth of MCL tumours in mouse xenograft models. In keeping with our in vitro data, Mino cells proliferated at a slower rate than Maver-1 cells, and therefore the effects of the drugs on proliferation were seen at a later stage [day 10 as opposed to day 7 (Fig. 3A,B)]. Both duvelisib and idelalisib treatment resulted a reduction of the size of tumours generated by both Maver-1 (Fig. 3A,C,D) and Mino (Fig. 3B,E,F) as compared to the vehicle control; the inhibition of growth continued until day 25. However, the reduction in tumour volume induced by duvelisib was more significant at early time points (days 7–14 for Maver-1 and days 10–17 for Mino) then that induced by idelalisib (Fig. 3).

### Expression of PI3Kγ by MCL cells regulates the response to chemokines

**Figure 4:**
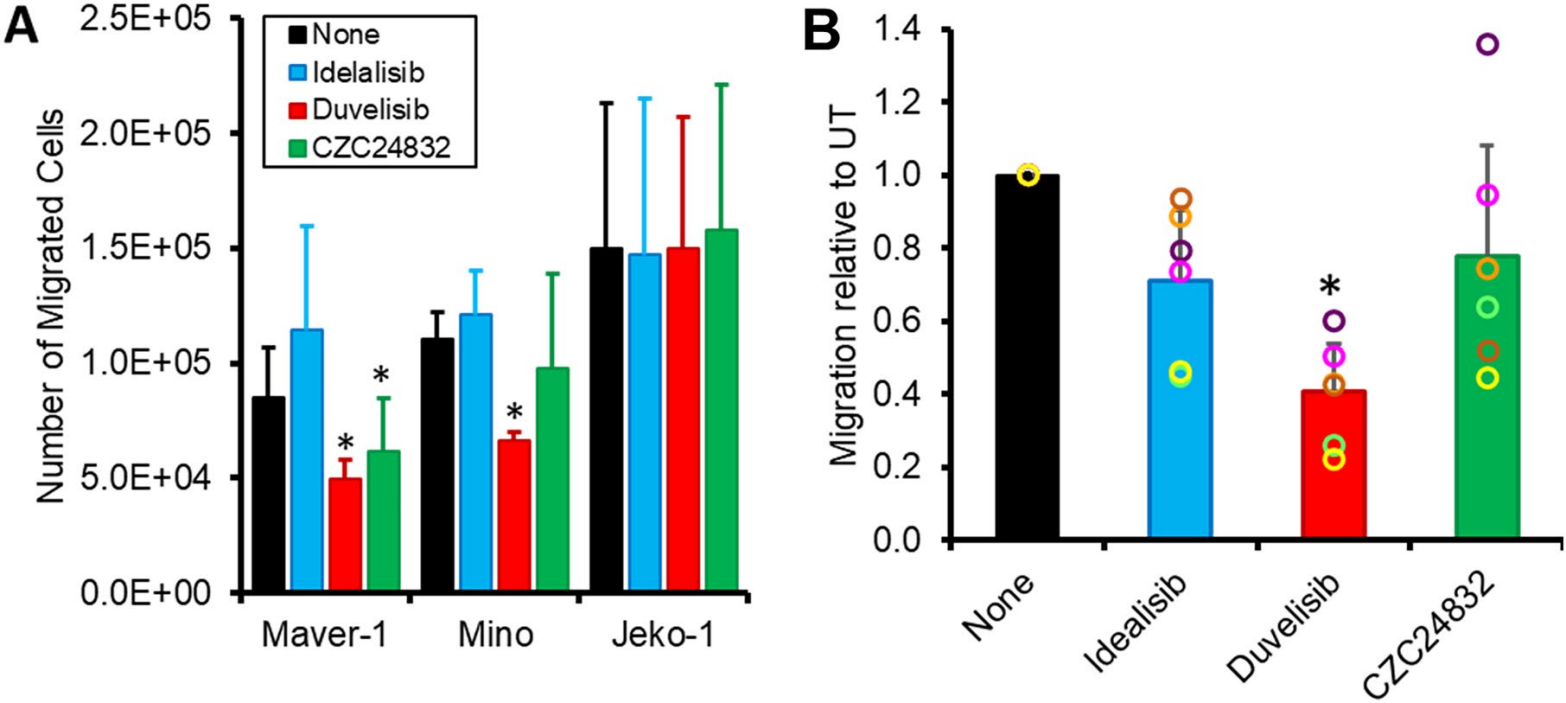
Inhibition of PI3Kγ reduces the motility of MCL cells. (**A**) Migration of Maver-1, Mino and Jeko-1 cell lines to CCL21 (1 µM) in the presence of the indicated inhibitors (mean ± SD of n = 3 separate experiments). (**B**) Migration of primary MCL cells to CCL21 in the presence of the indicated inhibitors (mean ± SD, n = 6 patients). Due to the variation in the number of cells migrating from the different patients’ data are normalised against migration of untreated cells to CCL21; circles represent the migration of cells from each of the individual patients (data for individual patients can be found in Supplementary Figure 4D). *P < *0.05.* Statistical significance was determined using the paired Student’s T-Test; for (**B**) the test was performed on the raw data.

We next investigated the role of PI3Kγ in migration, since the kinase has been shown to play an important role in mediating chemokine-induced signalling and cell migration in monocytes and neutrophils^30,37,38^, but not in B cells. We chose CCL21 as opposed to CXCL12 and CXCL13 as the migration of B cells into LN is absolutely dependent on this chemokine^39,40^. Whereas CXCL13 is involved in movement and adhesion within the follicles^40,41^ and MCL cells do not migrate into follicle but remain on the periphery; and CXCL12 is involved in BM adhesion and migration^42,43^. In keeping with the different roles of these chemokines, MCL cells migrated to CCL21 (Fig. 4) whereas they showed little or no migration to CXCL13 and CXCL12 (Supplementary Figure 4A, B). Pre-treatment of MCL lines with duvelisib significantly inhibited the migration of Maver-1 (*P* = 0.03) and Mino cells (*P* = 0.004), as did CZC24832 incubation on Maver-1 (*P* = 0.038). In contrast, idelalisib and A66 had no effect [(*P* > 0.157; n = 3); Fig. 4A and Supplementary Figure 4C]. None of the inhibitors had any effect on the migration of Jeko-1 cells [(*P* > 0.15, n = 3); Fig. 4A and Supplementary Figure 4C].

We next sought to confirm these observations using MCL PBMC. Although the number of MCL cells migrating in response to CCL21 differed greatly between patients, pre-treatment with duvelisib significantly and substantially (> 40%) inhibited MCL lymphocyte migration in every case [n = 6; Fig. 4B and Supplementary Figure 4D; (*P* = 0.02)]. In contrast, inhibition in response to CZC24832 and idelalisib was only seen in some patients (3 and 2 respectively) and was not significant when taking the patient group as a whole (*P* > 0.06). Treatment with A66 had no effect [*P* = 0*.*22; (Supplementary Figure 4D)]. Since inhibition of either PI3Kγ or PI3Kδ had no effect, when taken together these results indicate that PI3Kγ co-operates with PI3Kδ to mediate the chemokine-induced migration of MCL cells.

### PI3K is not required for the adhesion of MCL cells

We next investigated the potential roles of PI3K on MCL cell adhesion since the growth and survival of MCL cells is dependent on adhesion in the permissive microenvironment of the LN^9,17,23^. Retention of MCL cells within the LN microenvironment involves α4 integrin and the chemokine CXCL13^44,45^; both PI3Kγ^29^ and PI3Kδ^46^ regulate α4-mediated adhesion in other leukocytes. However, PI3K inhibitor treatment of MCL cells did not have any significant effect on adhesion of either MCL cell lines (Supplementary Figure 5A) or primary MCL cells to VCAM-1 (n = 2*;* Supplementary Figure 5B). Thus, the adhesion of MCL cells does not require PI3K signalling.

## Discussion

**Figure 5:**
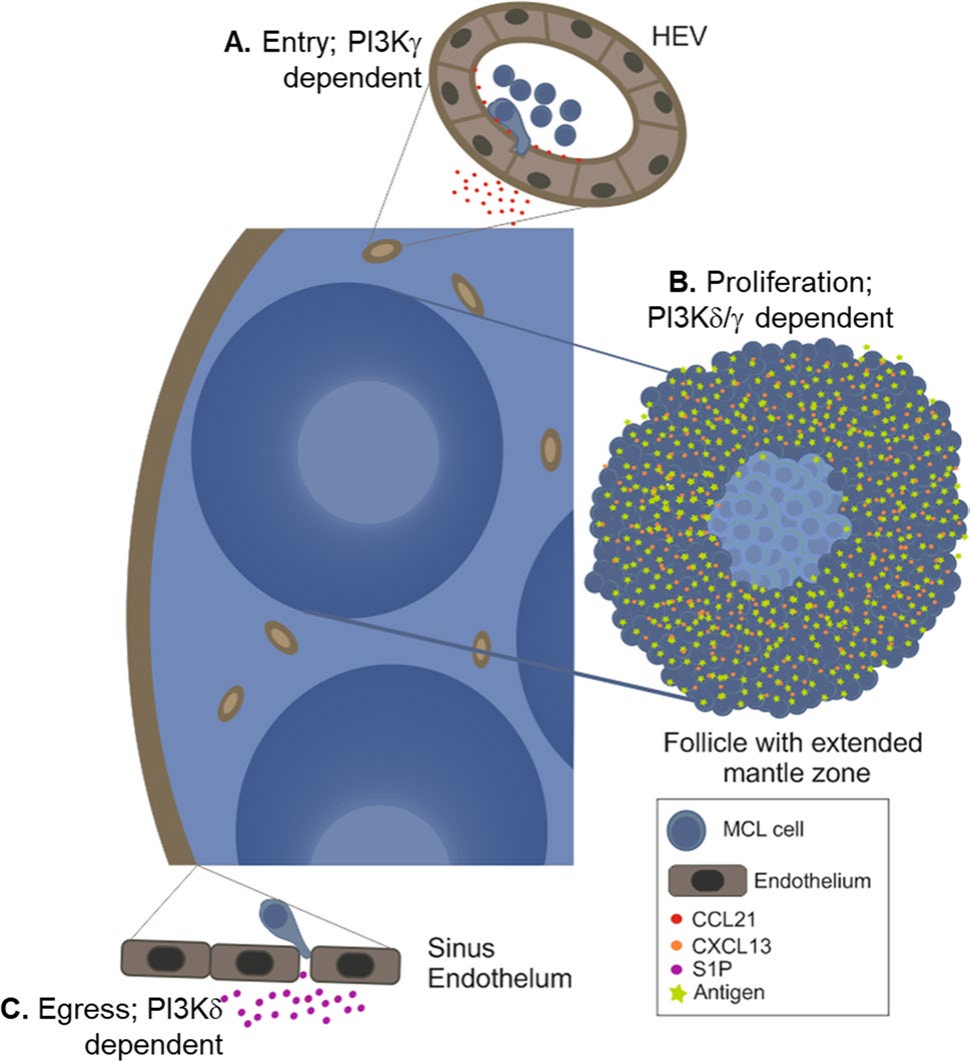
Schematic diagram of a MCL LN demonstrating the role that PI3K plays in the stages which determine lymphadenopathy. (**A**) Lymphocytes enter into the LN via the high endothelial venule (HEV) in response to CCL21 and move towards the follicle in a CXCL13-dependent manner; Chemokine-directed motility is dependent on PI3Kγ. (**B**) Once in the follicle they encounter antigen, undergo clonal expansion and are retained in the LN; proliferation is dependent on PI3Kδ/γ. (**C**) Exit (egress) is through the sinus endothelium is dependent on S1P. Expression of the egress receptor S1PR1 is inhibited by BCR signalling through PI3Kδ. Drawn using CoralDRAW 2017 (https://www.corel.com/en/).

In this study, we show that PI3Kγ is expressed by MCL cells and impacts multiple points of the disease process that are important for disease progression (Fig. 5). Thus, our data suggests a model of MCL where PI3Kγ is important for tumour proliferation and a combination of both PI3Kδ and PI3Kγ are important for cell migration. Overall, our data supports dual inhibition of PI3Kδ and PI3Kγ with duvelisib as a potentially effective treatment option for this incurable malignancy.

Migration into, between and within, SLO is important for cells to reach the LME of mantle zone where the cells grow and survive^9,23^. PI3Kγ has been previously demonstrated to be important for the migration of both T cells and monocytes^30,37,38^. Herein, we demonstrate that this kinase is also important for the migration of MCL cell lines and PBMC (Fig. 5A). In line with the fact that there is known to be functional redundancy between the different isoforms of PI3K^47^, inhibition of both PI3Kγ and PI3Kδ was required in order to reduce MCL cell migration. A role for PI3Kγ in CLL-cell migration has also been described^48^, however intriguingly inhibition was seen with idelalisib and CZC24832, but not duvelisib; the authors did not discuss the reasons for this apparent discrepancy.

In addition to being involved in trafficking to the LME, we demonstrated that PI3Kγ is important for the proliferation of MCL cells (Fig. 5B). As with migration, dual inhibition of PI3Kδ/γ was necessary to reduce the proliferation of MCL cells. The inhibition of proliferation is not due to cell cycle arrest, or cell death, but the result of a reduced rate of proliferation. This effect was evident both in vitro, and in vivo in our mouse xenograft models.

Although PI3Kγ is involved in the polarisation of macrophages, and inhibition of the kinase has been shown to reduce the growth of solid tumours^49,50^, our in vitro data suggest that the inhibition of MCL proliferation we see our in vivo experiments is mainly due to a direct effect on the MCL cells themselves. Our data is in line with the fact that inhibition of mTOR and AKT, downstream targets of PI3K, have been shown to result in decreased proliferation of MCL cells in vitro^51^. Thus, PI3Kδ/γ inhibition of proliferation may be mediated through the role of mTOR in the regulation cellular metabolism and protein synthesis^52^, which are required for cell division. However, our results differ from those described by Yang et al^53^, who demonstrated that inhibition of PI3Kδ alone resulted in a minor reduction in the proliferation and survival of Jeko-1 cells; although consistent with our data they showed no effect on Mino cells. The reason for this discrepancy is unclear, however it may be in part due to the higher dose of idelalisib used in their experiments (5 μM), which is approximately five times higher than the peak plasma level achieved in patients receiving treatment with the inhibitor^33^.

In addition to the role that PI3Kδ and PI3Kγ together play in the proliferation of MCL cells, PI3Kδ signalling, activated through the BCR, plays an important role in the pathogenesis of many B-NHL^8^. One of the roles of PI3Kδ in B-NHL is to regulate SIPR1; expression of this receptor regulates egress (exit) from LN. Inhibition of PI3Kδ with idelalisib results in expression of S1PR1 and migration to its ligand S1P^54^. Thus, we have previously shown that inhibition of PI3Kδ signalling facilitates exit of both normal and B-NHL cells from the LN. This data suggests that duvelisib treatment would also remove MCL cells from protective microenvironment of the LN into the circulation (Fig. 5C) and thus render the cells more sensitive to treatment with other therapeutics^54^.

We did not find a role for PI3Kα in the migration, adhesion, survival, or proliferation of MCL cells although this isoform is overexpressed in the majority of cases of MCL and has been implicated in resistance to idelalisib^36^. The dual PI3Kα/δ inhibitor copanlisib has been approved for use as a single agent in the treatment of FL^55^, as well as being shown to be active MCL in vitro^56^. However, since copanlisib is a relatively non-specific PI3K inhibitor, it is unclear which isoform of the kinase is mediating the response (IC50 for PI3Kα 0.3 nM; PI3Kδ 0.7 nM PI3Kγ 6.7 nM; PI3Kβ 3.4 nM).

PI3Kγ is also involved in MCL resistance to BTK where treatment failure has been demonstrated to be due to kinase reprogramming including increased expression of PI3Kγ^35^. As a results, patients who become refractory to ibrutinib have a dismal prognosis, regardless of the follow-on treatment used^57^; although duvelisib has not been tried in this context. Kinase reprogramming leading to patients becoming refractory to other treatment options is also seen in solid tumours, where it has been suggested that that combination strategies using 3, but not 2, drugs/inhibitors will be more effective when deployed upfront rather than as salvage treatment^58^. With regard to duvelisib has been used in a trial basis on B-NHL, however there were not enough patients with MCL included in the study to make any conclusion as to its efficacy^31^. However, the drug has been approved for the closely related B-NHL CLL as third-line therapy^59^. The data we present here suggest that duvelisib could be useful as part of a combination strategy in the treatment of MCL; this approach is currently undergoing clinical trials in CLL^60^. It is also important to note, in light of the fact that aberrations of *TP53* result in inferior responses to therapy^61^, that duvelisib reduced the proliferation and migration of both the Maver-1^62^ and Mino^63^ cell lines which have mutations in *TP53*.

In conclusion, our data demonstrate that PI3Kγ and PI3Kδ play important roles in processes critical for MCL pathophysiology and therefore suggest that duvelisib would provide an effective treatment for MCL patients, including those patients with *TP53* aberrations, a patient group for whom there are few treatment options. We therefore propose that the use of duvelisib as part of combination therapy, is examined in MCL in the clinical trial setting.

## Patients and methods

### Patients and cell lines

Patient samples were obtained with informed consent and with the approval of the Liverpool Research Ethics Committee. All experiments using primary MCL cells were approved by LHP SPARK Non-Interventional Sponsorship Sub Committee. Formalin fixed, paraffin embedded tissue samples were obtained from the SLO of 27 patients with B-NHL [9 CLL/small lymphocytic leukaemia (SLL), 10 FL, 8 diffuse large B cell lymphoma (DLBCL), 4 MCL, 3 splenic marginal-zone lymphoma (SMZL) and 2 nodal MZL]*.* Peripheral blood mononuclear cells (PBMC) were obtained from 6 MCL patients with blood involvement. Clinical data linked to the MCL samples are shown in Supplementary Table 1.

The MCL cell lines Maver-1, Mino and Jeko-1 and the HS-5 stromal cell line (DMSZ, Germany) were used.

### Tissue microarrays (TMA) analysis

A standardised scoring method was used for TMA analysis. The scores for the percentage of positive lymphocytes were as follows: none (0), 1–24% (1), 25–49% (2), 50–74% (3) and 75–100% (4). For IHC the intensity of the stained lymphocytes was scored as weak (1), moderate (2) or strong (3); for RNAscope intensity was defined by the number of positive dots/cell—none (0), 1–3 (1), 4–10 (2), > 10 (3). The total score = percentage score × intensity score. A minimum of two cores were evaluated for each patient by two independent researchers, and the scores were averaged. A patient was considered to express PI3Kγ in their SLO when ≥ 50% of the cells were positive and the mean intensity was ≥ 1.5.

### Antibodies

Antibodies to the following targets were used: PI3Kγ (ab70912; Abcam, Cambridge UK); PI3Kδ (SC-55589; Santa Cruz Biotechnology, Heidelberg, Germany); CD19, CD105, CCR7, CXCR4, CXCR5 (BD, Oxford); CD20 (Dako, Cheedle).

### Immunohistochemical (IHC) staining

For the TMA, the EnVision™ staining method was used (Dako, Cambridgeshire). Slides were then stained for PI3Kγ, CD20 and PI3Kδ on adjacent sections using DAB (brown) as the chromogenic substrate. Slides were then counter stained with haematoxylin (blue).

### In situ hybridisation

RNAscope staining was performed according to the manufacturer’s instructions (ACD Bio, Italy); using probes to PIK3CG (590,471) together with a positive (PPIB) and negative control. Cores where the positive control showed no staining were excluded from analysis; the negative control did not stain in any of the tissue sections.

### Flow cytometry

Cells were stained by direct immunofluorescence and analysed by flow cytometry (Attune NxT; ThermoFisher, Paisley).

### Western blotting

The expression of PI3Kγ (p110γ) on the MCL cell lines and PBMC from MCL patients was analysed by western blotting. Primary cells were purified by MACS (Miltinyi Biotech, Woking) to remove non-B cells as T cells and monocytes are known to express PI3Kγ (> 99% purity; data not shown). The monocyte cell line THP-1 was used as a positive control for PI3Kγ expression.

In order to determine the efficacy of the PI3K inhibitors MCL cells were incubated for 1 h with inhibitors to different PI3K isoforms [PI3Kα (A66), PI3Kδ (idelalisib), PI3Kγ (CZC24832) (all from Selleck, Cambridge UK), and the dual PI3Kδ/γ inhibitor [(duvelisib) Verastem Oncology]. The effects of the inhibitors on the phosphorylation of AKT in response to BCR cross-linking with goat anti-human IgM [F(ab)2 fragments; 20 μg/ml; Jackson Immunoresearch, Pensylvania)] and chemokine signalling with CLL21 [(1 µM) Biotechne, Abbingdon] were then examined by western blotting. Inhibitors were titrated using the following concentrations 100 nM; 500 nM; 1 μM and 2 μM. AKT phosphorylation was inhibited by 1 μM (Supplementary Figure 1F) which is below the peak plasma concentration for idelalisib and duvelisib^33,34^.

### Proliferation assay; cell lines

Maver-1, Mino and Jeko-1 cells were incubated for 24, 48 and 72 h with inhibitors to the PI3K isoforms. Proliferation was then measured using the ClickIT™ EDU (5-ethynyl-2´-deoxyuridine) method (ThermoFisher), according to the manufacturer’s instructions. Briefly, 10 μM EDU was added for the last 4 h of the incubation. Cells were then harvested, fixed and permeabilised, before incubation with the ClickIT™ EDU reaction cocktail. Finally, the DNA binding dye 7′AAD (ThermoFisher) was added in order also to measure the cell cycle phase. The proportion of cells which were proliferating, as well as the cell cycle stage, were then analysed by flow cytometry. The number of cells was also counted manually.

### Proliferation assay; primary cells

Since primary mantle cells undergo rapid apoptosis in vitro when cultured in the absence of stromal cell support^19^, MCL cells stained with CFSE (ThermoFisher) were cultured with irradiated cells stromal cells (HS-5) in the presence of IL-4 (10 ng/ml; ThermoFisher) together with PI3K inhibitors. Every 4 days half the medium was removed and replaced with fresh medium containing IL-4 plus the appropriate PI3K inhibitor. MCL cells were harvested at days 7, 10 and 14 and the MCL cells were stained with CD19 and HS5 cells with CD105. The percentage of MCL cells (CD20^+^CD105^-^) which had decreased CFSE staining was then measured by flow cytometry.

### In vivo proliferation assays

All the procedures related to animal handling, care and the treatment in this study were performed according to the guidelines approved by the Institutional Animal Care and Use Committee (IACUC) of Shanghai Chempartner following the guidance of the Association for Assessment and Accreditation of Laboratory Animal Care (AAALAC). Moreover, the reporting of the data in the manuscript follows the recommendations in the ARRIVE guidelines. CB17 SCID mice were injected with 5 × 10^6^ Maver-1 or Mino cells subcutaneously in 50% Matrigel (0.2 ml mixture). When the tumours had reached 60–160 mm^3^ mice were sorted into 3 groups (10 mice/group) and treated by oral gavage with either vehicle (5% DMSO + 40% PEG400 + 55% water) PO BID, duvelisib 50 mg/kg PO BID or idelalisib 50 mg/kg PO BID. Tumour volume and body weights were measured twice weekly for the duration of the study. Tumour sizes (mm^3^) were measured with a calliper in two dimensions and calculated using the formula: (a × b^2^)/2. Where a = half of length and b = half of width of the tumours.

### Apoptosis assay

MCL cells were cultured for up to 72 h and cell death was measured using Anexin V-FITC (BD) and 7’AAD (ThermoFisher) staining followed by flow cytometry.

### Migration assay

MCL cells which had been incubated with inhibitors to PI3K isoforms were placed on the inserts of Transwell plates (5 μm pore size; Corning, High Wycombe). CCL21 (1 μg/ml) was added to the bottom wells at a concentration which had been shown to induce maximum migration (data not shown) and MCL cells were added to the inserts. After 6 h the number of (CD19^+^) B cells that had migrated to the bottom wells was counted. Since the migration of primary cells differed greatly between patients, migration of treated cells was normalised to migration in response to chemokine alone.

### Adhesion assay

MCL cells were stained with calcien-AM (10 μM; ThermoFisher) for 1 h. Cells were then washed prior to incubation with PI3K inhibitors, and then incubated for 2 h on plates which had been coated with VCAM-1 (1 μM) ± CXCL13 [(500 ng/ml) R&D]. After washing to remove non-adherent cells, fluorescence was measured on a plate reader (Polarstar Omega; BMG Labtech, Germany). The number of adherent cells was calculated with reference to a calibration curve.

### Statistical analysis

To determine whether there were any differences between the different subtypes of B-NHL on the TMAs the Krurskal-Wallis test was used, where statistical significance was suggested at the 0.05 level, Tukey’s method was used to correct for multiple comparisons. Post-hoc pair-wise comparisons were performed using the Mann–Whitney U-test where significance was maintained at the 0.05 level. Pearson’s correlation coefficient was used to analyse the relationship between TMA staining of protein (IHC) and mRNA (RNAscope). The paired Student’s T-test was used to determine if there were any statistically significant differences between the different PI3K inhibitors in the in vitro assays; the unpaired T-test was used in the mouse experiments.

## Supplementary Information

Supplementary Information 1.

Supplementary Information 2.

## Data Availability

There are no large datasets in this paper; original data for the figures is available, on request, from KJT.
