# Supplementary material for: Roles of PI3Kγ and PI3Kδ in mantle cell lymphoma proliferation and migration contributing to efficacy of the PI3Kγ/δ inhibitor duvelisib
Source: Sci Rep. 2023 Mar 7;13:3793. doi: 10.1038/s41598-023-30148-3 (PMC9992372; doi:10.1038/s41598-023-30148-3)
Supplement: Supplementary file 1 — Supplementary Information 1. [file 41598_2023_30148_MOESM1_ESM.pdf]

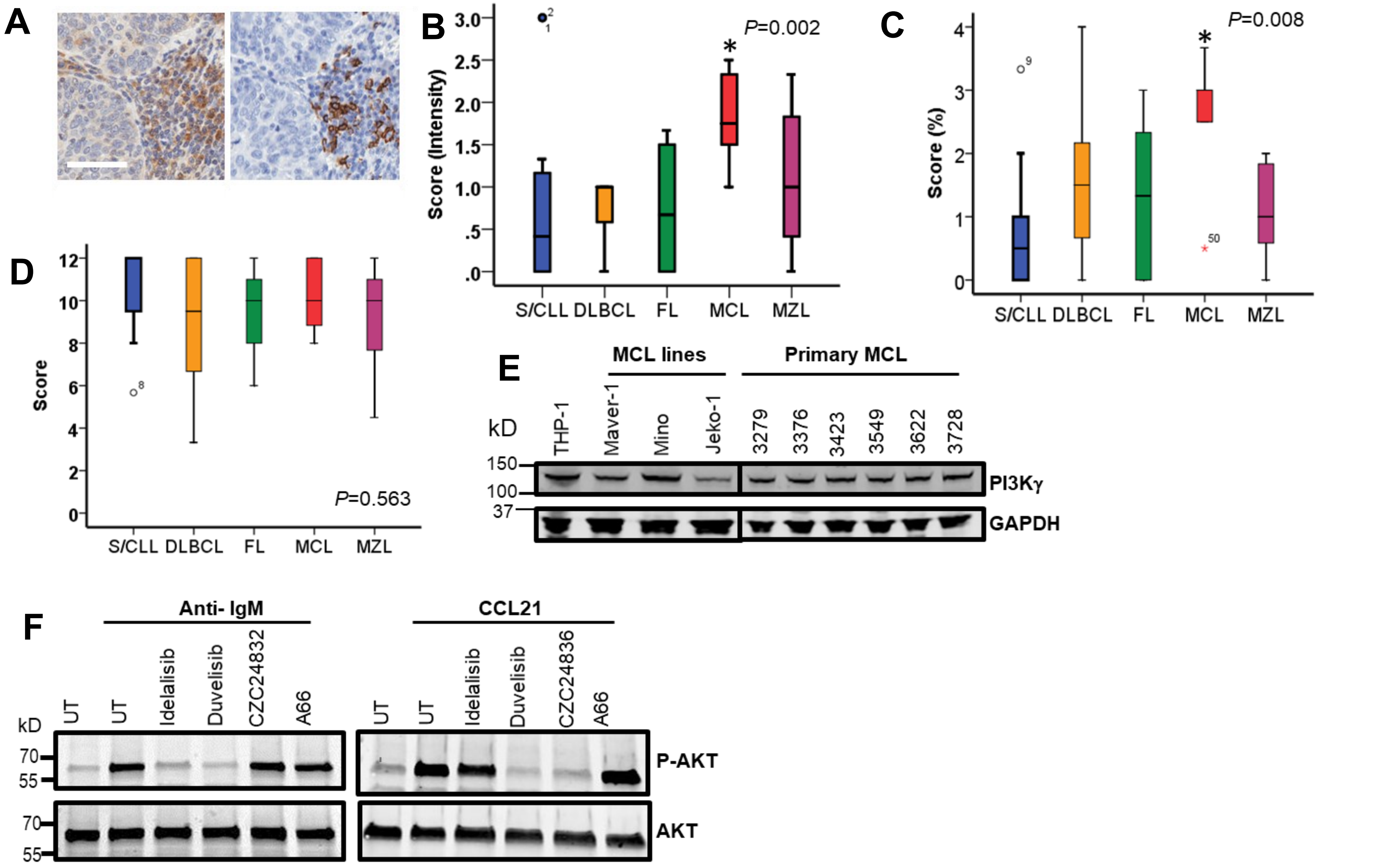

Figure 1. TMA data for PI3K receptor expression, and inhibitor titration. **A.** Tonsil control TMA tissue; left panel PI3Kγ; right panel CD20. Bar 50 μM. **B&C.** IHC TMA scores for **(B)** intensity and (**C)** percentage of positive B cells which were used to determine the total score shown in Figure 1B. **D.** The same TMA analysed for PI3Kδ. **E.** Western blot showing expression of PI3Kγ (p110) on MCL cell lines and primary cells (the monocyte line THP-1 was used as a positive control for receptor expression; GAPDH was used as a loading control). **F.** Maver-1 cells were treated with PI3K inhibitors (idelalisib, duvelisib and CZC24836 at 1 μM; A66 at 500 nM) prior to stimulation with either anti-IgM or CCL21 and a western blot was performed for ^{473}S-AKT phosphorylation and total AKT.

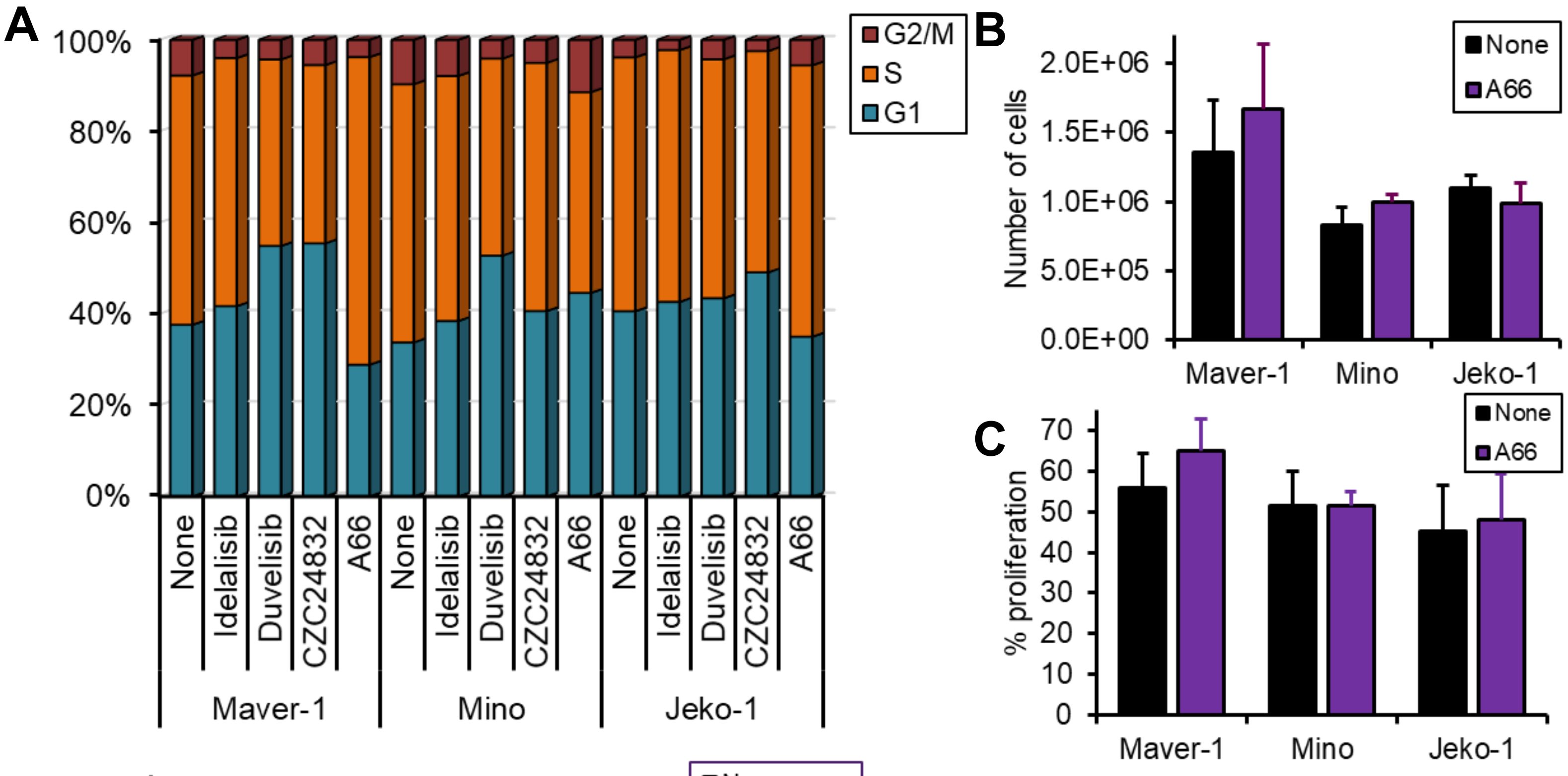

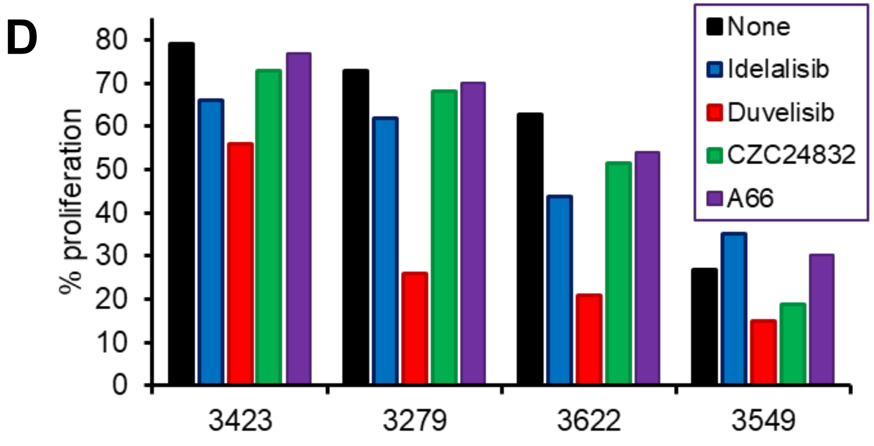

Figure 2. Effect of PI3K inhibition on cell cycle stage and proliferation. **A.** Cell cycle data was calculated using the ClickIT Edu assay; the combination of Edu and the cell cycle dye 7'AAD was used to determine the number of cells in each stage of the cell cycle. The data is derived from the experiments shown Figure 2A. Data is shown as mean cell cycle stage. Although it can be seen that there are less cells in cycle in Maver-1 with duvelisib and CZC24832 treatment, and in Mino with duvelisib treatment this did not reach statistical significance ($P>0.05$) **B & C** Effects of PI3Kα inhibition on MCL cell lines **(B)** the number of cells **(C)** Proliferation (data derived from the experiments in Figure 3A). **D.** Proliferation of primary MCL PBMC (raw data for Figure 2C).

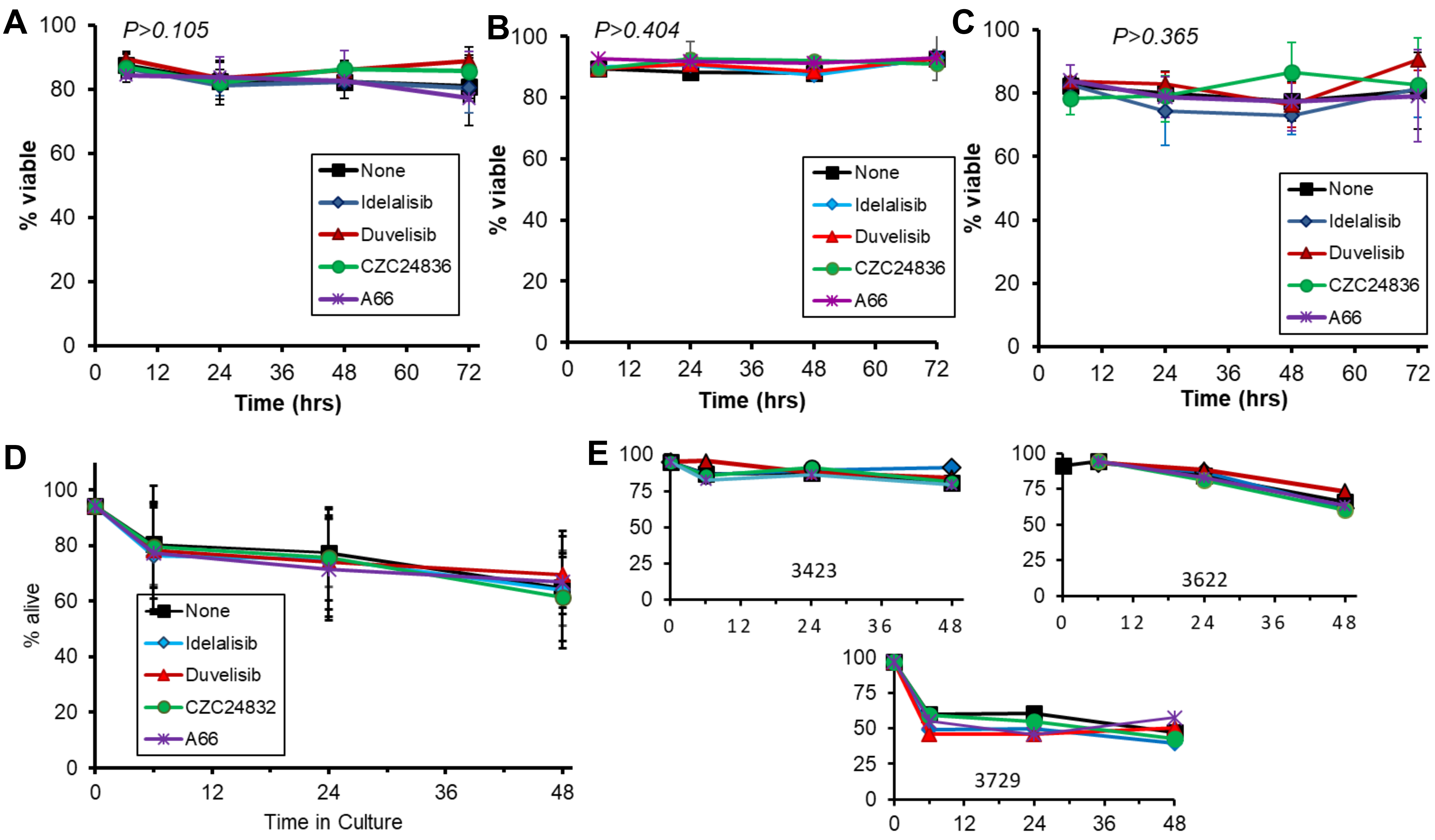

Figure 3. Effect of PI3K on apoptosis. MCL cells were cultured for up to 72 hrs; apoptosis was measured using Annexin V/7AAD staining and flow cytometry **(A)** Maver-1**. (B)** Mino **(C)** Jeko-1. Data is mean ± SEM of 3 separate experiments. (**D)** Primary MCL cells cultured on an HS-5 stromal cell feeder layer in the presence of IL-4 (n=3) **E.** Individual patient data for (D). Statistical significance was determined using the paired Student's T-test.

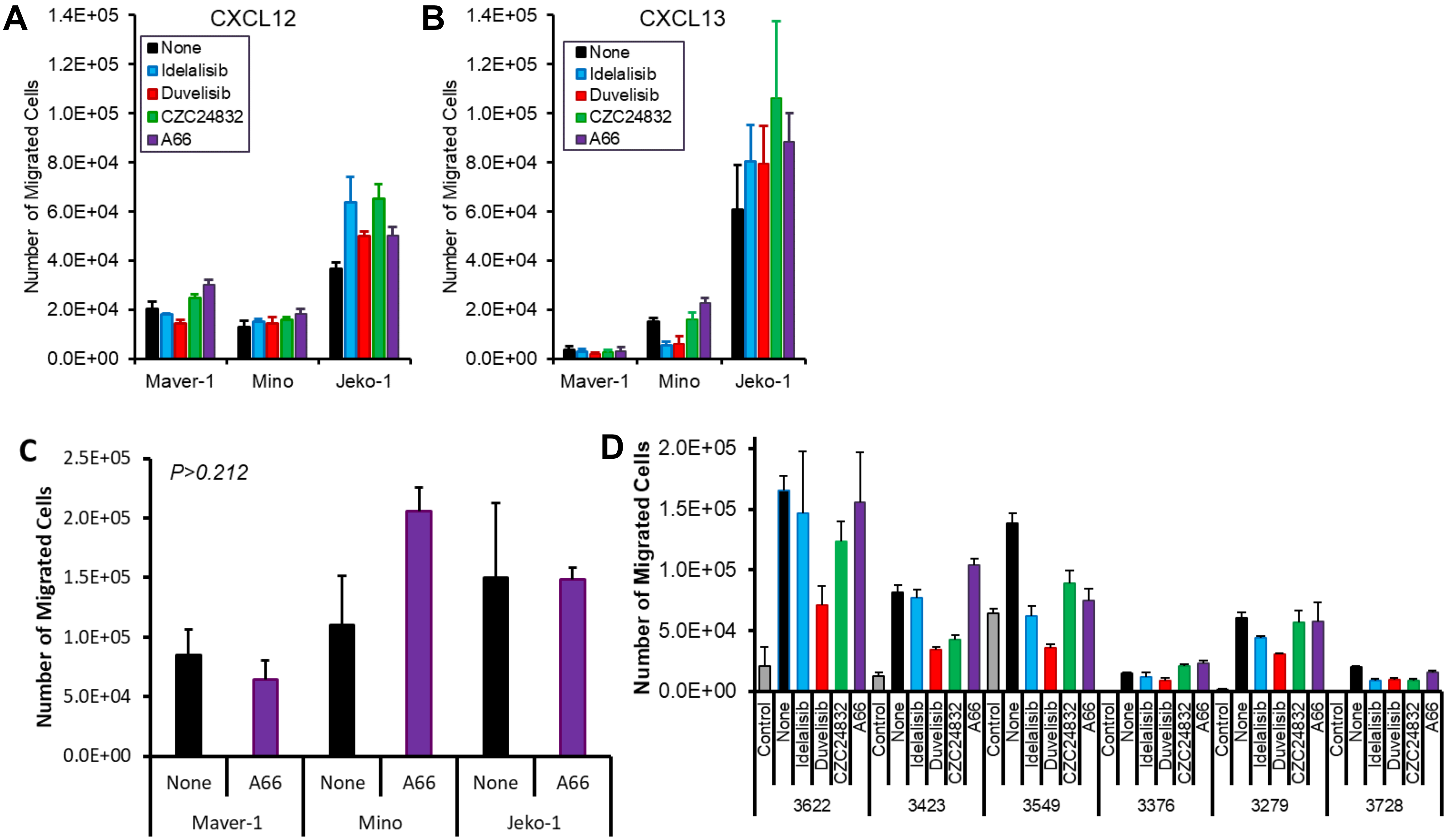

<u>Figure 4. Effect of PI3K inhibition on MCL migration.</u> **A.** Migration of MCL cell lines to CXCL12 and **(B)** CXCL13 (mean ± SD of n=3 separate experiments). **C.** Effects of PI3Kα inhibition on migration of cell lines (mean ± SD of n=3 separate experiments – data was from the same the experiments shown in Figure 4A). **D.** Raw data for Figure 4B showing effects of PI3K inhibitors on primary cells from individual patients (mean ± SD of 3 technical replicates). Paired Student's T-test was used to determine statistical significance.

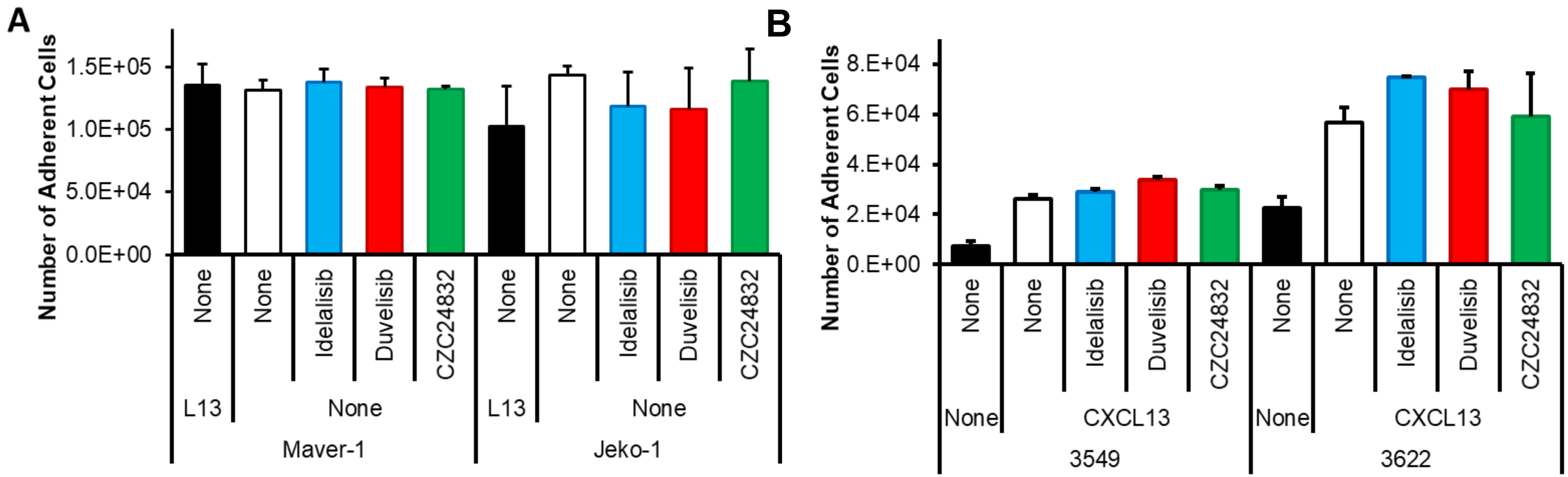

<u>Supplementary Figure 5. Effect of PI3K inhibition on MCL cell adhesion to VCAM-1.</u> CXCL13 (L13) 500 ng/ml was used to induce adhesion. **A.** Maver-1 and Jeko-1 cells were constitutively adherent to VCAM-1 so the effect of inhibitors was analysed in the absence of CXCL13. (Mean ± SD of n=3 separate experiments). P>0.18 relative to the UT control. **B**. CXCL13 was required in order for primary MCL cells to adhere to VCAM-1 so the effect of inhibition in the presence of chemokine is shown. (Mean ± SD of n=3 technical replicates for each patient case used). P>0.08 relative to the UT control. Paired Students T-test was used to determine statistical significance.

A

Patient no	Age	Sex	Treatment	Status
H06-18190	70	M	R-CHOP	Dead (2008)
H05-20780	61	F	Chemotherapy (NA) followed by allo-BMT	Dead (2006)
H06-17558	78	F	None (refused)	Dead (2011)
H05-13516	79	M	Excision; L-CHOP	Dead (2007)
H04-16991	62	M	Allograft	NA
H06-01573	75	M	NA	NA

B

Patient no	Age (DOB)	Sex	Stage	Date of diagnosis	Treatments (dates)	Disease status	Alive (date)	Dead (date)
3279	NA	NA	NA	NA	NA	NA	NA	NA
3423	22.12.58	M	IVa	25.07.2014	None	PD 09/2017	4.11.19	
3376	13.11.93	M	IV		3.7.14 (R+B+ibrutinib)<br>21.10.14 (velcade + prednisolone)			13.2.15
3549	10.12.21	M	IVa	2004	17.10.16 R-chlorambucil- little response,<br>April 2017 - ibrutinib (suspended AF) -restarted 31.10.2017-07.2018			12.2.19
3622	22.12.58	M	IVa	25.07.2014	Jan 2015 BEAM (Radiotherapy)- CR,<br>Mar 2015 Transplant<br>Nov 2017- to date Ibrutinib	PD 09/2017	4.11.19	
3728	10.04.36	M	III	10.06.2014	May 2019—rituximab and ibrutinib (ENRICH trial)<br>Maintenance rituximab (due to COVID-19) but ibrutinib restarted August 2020		8.10.20	

Supplementary Table S1. Clinical details of MCL patients. A. Tissue samples. B. Peripheral blood. CHOP cyclophosphamide, doxorubicin, vincristine, and prednisone; L-L-argenine Allo-BCT – allogeneic bone marrow transplant. R– rituximab; B – bendamustine. NA – data not available.
