# Supplementary material for: Roles of PI3Kγ and PI3Kδ in mantle cell lymphoma proliferation and migration contributing to efficacy of the PI3Kγ/δ inhibitor duvelisib
Source: Sci Rep. 2023 Mar 7;13:3793. doi: 10.1038/s41598-023-30148-3 (PMC9992372; doi:10.1038/s41598-023-30148-3)
Supplement: Supplementary file 2 — Supplementary Information 2. [file 41598_2023_30148_MOESM2_ESM.pdf]

D

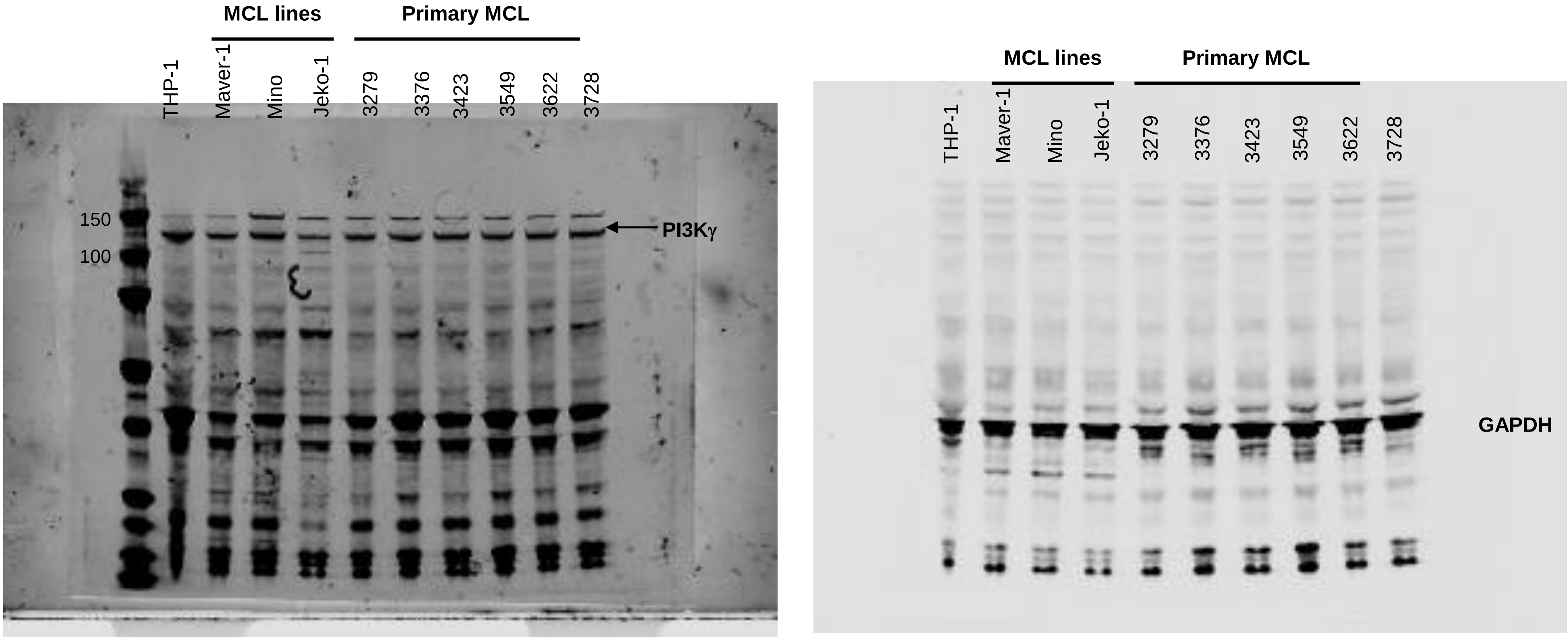

Full length blots for Supplementary Figure 1E.

F

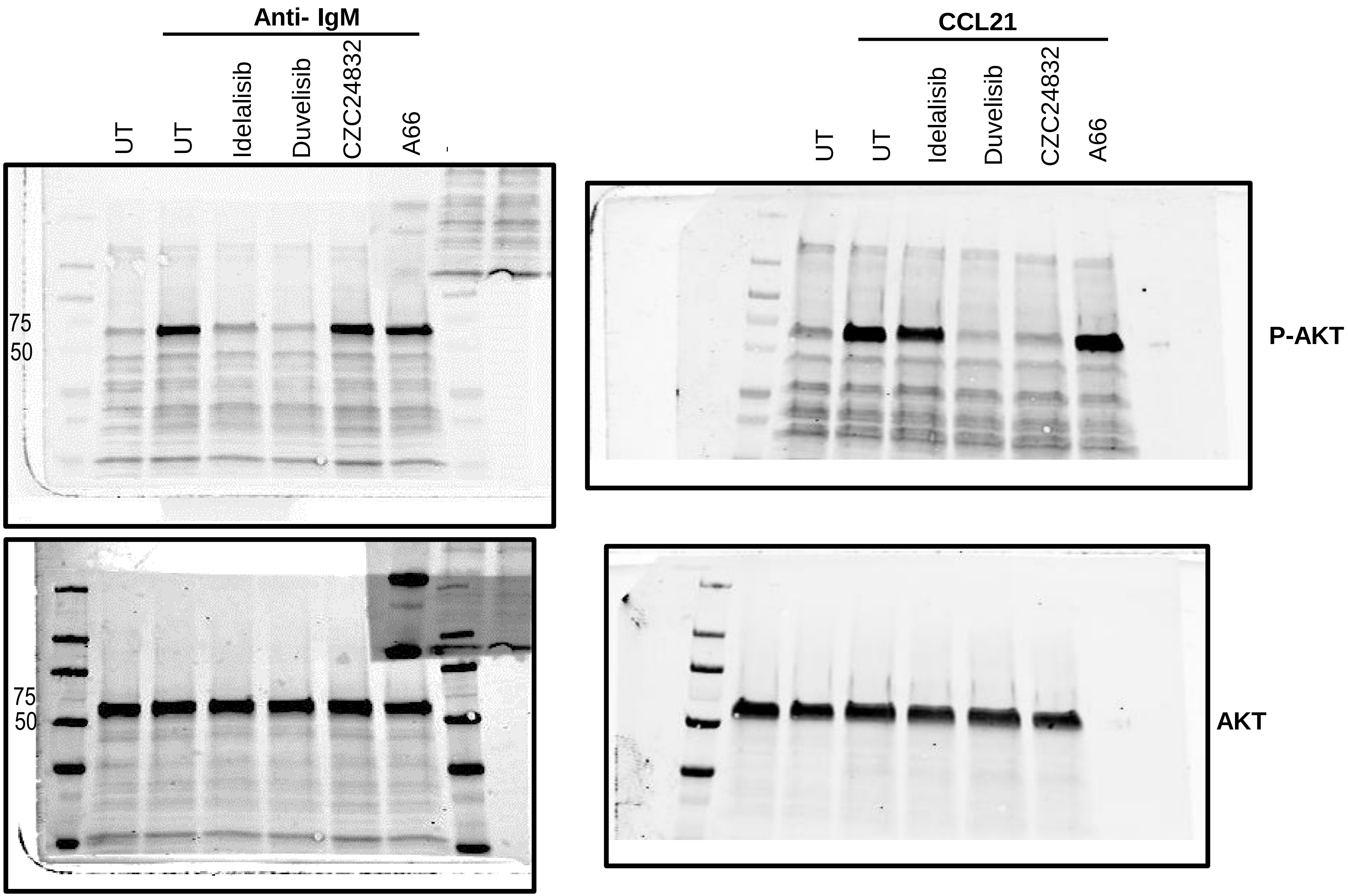

Full length blots for Supplementary Figure 1F. In the anti-IgM blot the corner of another blot being imaged at the same time can be seen in the top right corner. In the CCL21 blot image acquisition was stopped before the last millimetres of the gel had been acquired.
